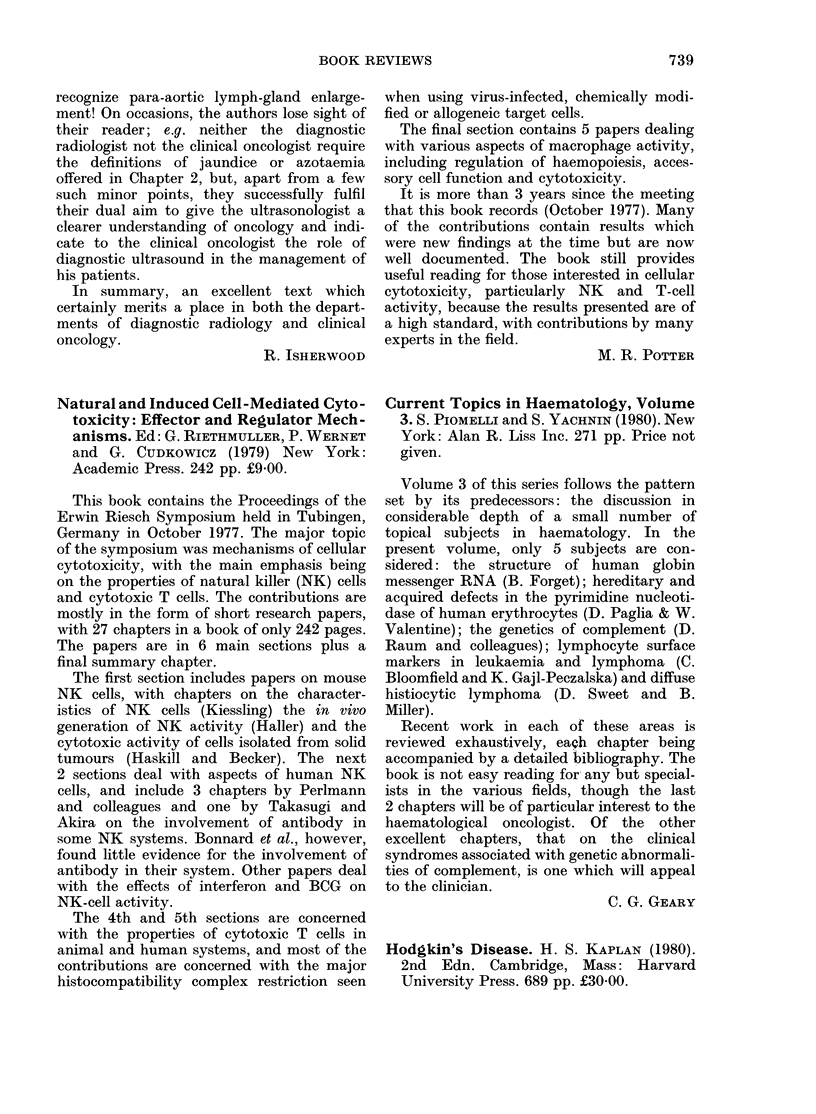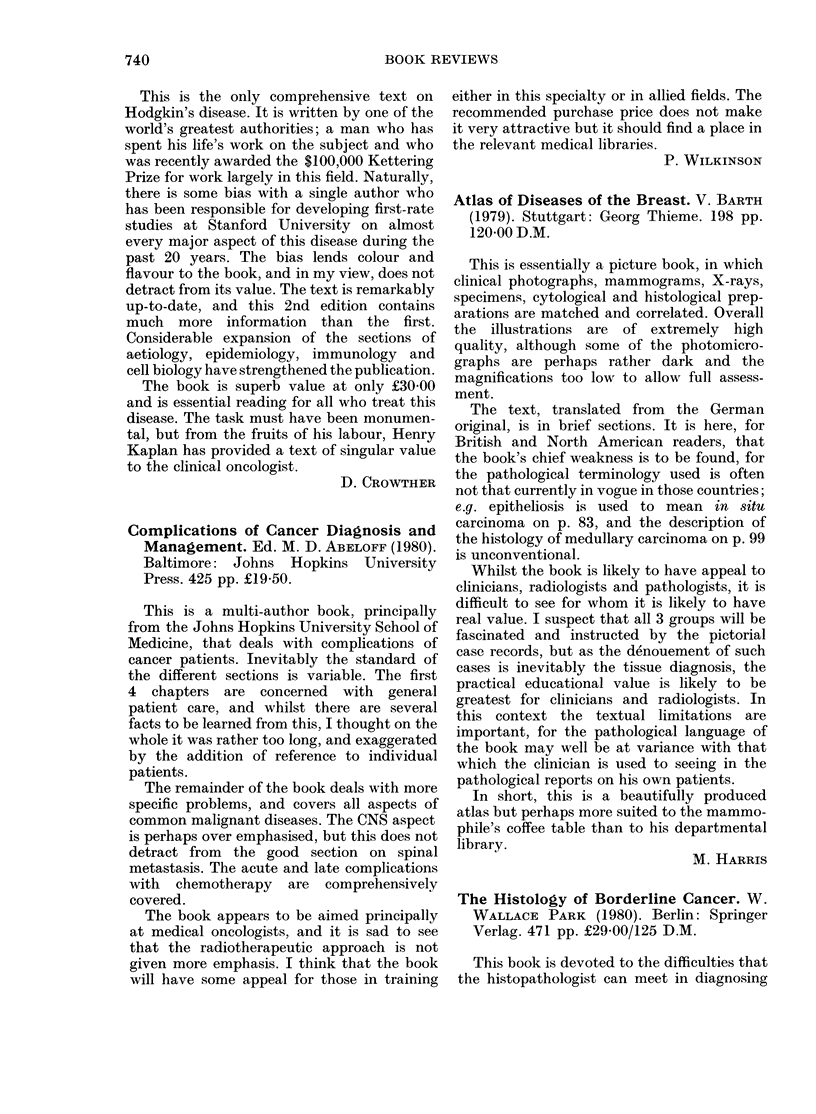# Hodgkin's Disease

**Published:** 1981-05

**Authors:** D. Crowther


**Hodgkin's Disease.** H. S. KAPLAN (1980). 2nd Edn. Cambridge, Mass: Harvard University Press. 689 pp. £30·00.

This is the only comprehensive text on Hodgkin's disease. It is written by one of the world's greatest authorities; a man who has spent his life's work on the subject and who was recently awarded the $100,000 Kettering Prize for work largely in this field. Naturally, there is some bias with a single author who has been responsible for developing first-rate studies at Stanford University on almost every major aspect of this disease during the past 20 years. The bias lends colour and flavour to the book, and in my view, does not detract from its value. The text is remarkably up-to-date, and this 2nd edition contains much more information than the first. Considerable expansion of the sections of aetiology, epidemiology, immunology and cell biology have strengthened the publication.

The book is superb value at only £30·00 and is essential reading for all who treat this disease. The task must have been monumental, but from the fruits of his labour, Henry Kaplan has provided a text of singular value to the clinical oncologist.

D. Crowther